# Altered Intrinsic Brain Activity in Patients With Late-Life Depression: A Resting-State Functional MRI Study

**DOI:** 10.3389/fpsyt.2022.894646

**Published:** 2022-05-23

**Authors:** Chaomeng Liu, Weigang Pan, Dandi Zhu, Peixian Mao, Yanping Ren, Xin Ma

**Affiliations:** ^1^The National Clinical Research Center for Mental Disorders & Beijing Key Laboratory of Mental Disorders, Beijing Anding Hospital, Capital Medical University, Beijing, China; ^2^Advanced Innovation Center for Human Brain Protection, Capital Medical University, Beijing, China

**Keywords:** intrinsic brain activity, late-life depression, percent amplitude of fluctuation, receiver operating characteristic, biomarker

## Abstract

**Objectives:**

To investigate the altered intrinsic brain activity (IBA) in patients suffering from late-life depression (LLD) using a percent amplitude of fluctuation (PerAF) method.

**Methods:**

In total, fifty patients with LLD and 40 non-depressed controls (NCs) were recruited for the present research. Participants underwent the Repeatable Battery for the Assessment of Neuropsychological Status (RBANS) test and resting-state functional MRI (rs-fMRI) scans. The RBANS test consists of 12 sub-tests that contribute to a total score and index scores across the following five domains: immediate memory, visuospatial/constructional, language, attention, and delayed memory. The PerAF method was used for data analysis to detect changes in neural activity in the relevant brain regions. A receiver operating characteristic (ROC) curve was conducted to evaluate the ability of the RBANS test and proposed the PerAF method in distinguishing the two groups. The relationships between altered IBA and neuropsychologic deficits were determined by the Pearson correlation analysis.

**Results:**

A significant difference existed in RBANS total score, immediate memory, visuospatial/constructional, language, attention, and delayed memory between groups (*P* < 0.05). Compared with the NCs group, the LLD group demonstrated decreased PerAF differences in the bilateral superior frontal gyrus, orbital part (Frontal_Sup_Orb), and bilateral anterior cingulate cortex (ACC). The PerAF method and RBANS test exhibited an excellent discriminatory power with the area under curve (AUC) values in distinguishing the two groups. In addition, the attention score of the RBANS test positively correlated with the PerAF values of the bilateral Frontal_Sup_Orb and bilateral ACC.

**Conclusion:**

The changes of PerAF in the bilateral Frontal_Sup_Orb and bilateral ACC are related to an increased risk of developing LLD. Moreover, the PerAF method could be used as an underlying sensitivity biomarker to identify the psychiatric disorder.

## Introduction

Depression after 60–65 is generally called late-life depression (LLD), affecting 4%−10% of the elderly (1, 2). Compared with the depression observed in the younger generation, LLD is often related to aging-associated neurodegeneration, cognitive impairment, or somatic complaints, increasing the risk for dementia, disability, and mortality (3). Exploring changes in the functional and structural connectivity in brain networks in association with emotional and cognitive symptoms may contribute to understanding the neural mechanisms underlying LLD, whereas the specific brain regions engaged with such changes are still unclear.

Recently, the modern brain neuroimaging techniques have developed rapidly. Functional magnetic resonance imaging (fMRI) has appeared as a popular technology because of its being non-invasive and does not require exposure to radioactive tracers, and provides new insights into the pathophysiology of depression. According to the collection status, fMRI is divided into task-state fMRI (ts-fMRI) and resting-state fMRI (rs-fMRI). In terms of the ts-fMRI study, hypoactivation of the anterior cingulate cortex (ACC) in the elderly patients experienced multiple depressive episodes in a verbal fluency task (4). However, another study did not observe the abnormal activation of ACC in response to an explicit sequence learning task, and they discovered diminished activation in the dorsolateral pre-frontal cortex (DLPFC) bilaterally while increased activation in the right caudate and putamen (5). Confounds associated with illness chronicity, such as the number of episodes and prolonged exposure to antidepressants, may be inconsistent across studies (6). Compared with the studies including patients with a long course of depression, few studies have investigated first-episode, treatment-naive patients with LLD. The study of the first-episode, drug-naive patients with LLD may be significant for elucidating the core pathogenesis of this illness. In addition, another issue pertains to the task-related functional neuroimaging studies that require patients to follow complicated cognitive tasks, and thus, the performance may confound the results (7).

Rs-fMRI has been considered a feasible and widely accepted method since the study of Biswal et al. (8). They first reported that the spontaneous low-frequency (0.01-0.08 Hz) fluctuations were closely associated with the intrinsic brain activity (IBA) and physiological meaning. IBA indicates sustained neural activity, which affects brain functions (9). In rs-fMRI studies of depression, most of the current work has focused on the major depressive disorder (MDD), regional brain activity in the frontal, temporal, occipital, and cerebellar lobes, and also in the thalamus and insula, displays reduced local synchronization among patients with MDD (10–12). The amplitude of low-frequency fluctuations (ALFFs), fractional ALFF (fALFF), and regional homogeneity (ReHo) constitute three major rs-fMRI approaches in testing IBA (13, 14). An rs-fMRI study using ALFF to investigate first-episode, drug-naive patients with LLD revealed that compared with the control group, the left superior temporal gyrus activation increased while the activation of the bilateral superior frontal gyrus decreased (15). However, another study using ReHo demonstrated that the left Crus I of the cerebellum increased while the activation of the right precuneus decreased (16). The population criteria of the two studies aforementioned are similar, and the inconsistent results may be related to the fact that ALFF and ReHo methods are susceptible to high-frequency physiological cardiac and respiratory noises (17). As a novel approach, percent amplitude of fluctuation (PerAF) exhibits optimal performance in-degree centrality, ALFF, and regional homogeneity (18). Thus, the PerAF approach makes it possible to enhance sensitivity and lower bias while dealing with the IBA changes related to LLD. However, there is still a lack of research on LLD.

Research demonstrates that the severity of depressive symptomatology among patients with LLD contributes to neurocognitive decline (19), while the latest cross-sectional and longitudinal surveys have not recognized any prominent correlation between depression symptoms with cognitive impairments (20–22). Therefore, we hypothesized that compared with non-depressed controls, widespread IBA alternations occurred in patients with LLD. These changes were associated with depression symptoms or cognitive impairment, providing a good understanding of the neurobiological mechanisms that underlay LLD.

## Materials and Methods

### Participants

The present research recruited 50 patients suffering from LLD and 40 non-depressed controls (NCs) between February 2021 and October 2021. All the participants presented informed consent. The study protocols gained approval from the institutional review board of the Beijing Anding Hospital. All the subjects were 60–75 years old and right-handed, first episode and no previous treatment with psychotropic drugs, confirmed by a certified geriatric psychiatrist through Axis I major depressive episode according to the 5th edition of Diagnostic and Statistical Manual of Mental Disorders (DSM-V) through the diagnostic interview (23, 24). Besides, no cases were diagnosed with additional Axis I major psychiatric disorders, except for anxiety disorders. Patients enrolled in the study had the least 17-item Hamilton depression rating scale (HAMD) (17). In addition, the participants were all requested to get the least Mini-Mental State Examination (MMSE) scale scores of 24 (excluding the presence of dementia) (25). Patients conforming to the following standards were excluded, including major neurocognitive decline, major head trauma history, Parkinson's disease, stroke, serious cardiovascular, respiratory, immune, and other systemic diseases.

### Cognitive Assessment

This study performed a cognitive assessment with the Repeatable Battery in the Assessment of Neuropsychological Status (RBANS) by well-trained clinicians (26). It contains 12 standardized cognitive tests categorized into the 5 following fields: visuospatial/constructional (line orientation and figure copy), immediate memory (story memory and list learning), attention (digit symbol coding and digit span), language (semantic fluency and picture naming), and delayed memory (list recognition, list recall, figure recall, and story recall). A higher RBANS score suggests superior cognitive performance. Former research has proved RBANS as a helpful screener to evaluate cognitive impairments in psychiatric patients (27).

### Rs-fMRI Protocol and Data Analysis

Each participant was scanned using the Siemens 3T scanner (Siemens, Erlangen, Germany), and the head was tightly fastened using foam pads and straps to avoid motion. This study initially captured images in T1 image (excluding intracranial organic diseases preliminary, such as tumor-like lesion or absence), and then the rs-fMRI was made (around 7 min). When collecting resting-state fMRI data, each participant was asked to relax with eyes closed, lie still, and keep awake. The T1 images was gathered with the T1-weighted sagittal 3D magnetization-prepared rapid gradient echo (MPRAGE) sequence: TE = 1.85 ms; TR = 2530 ms; FOV = 256 × 256 mm^2^; FA = 9°; voxel size = 1.0 × 1.0 × 1.0 mm^3^; thickness = 1.0 mm and matrix size = 256 × 256. Then, images were gathered in resting-state axially with the echo-planar imaging (EPI) sequence under the following parameters, echo time (TE) = 30 ms; repetition time (TR) = 2,000 ms; field of view (FOV) = 256 × 256 mm^2^; flip angle (FA) = 90°; axial slices = 33; matrix size = 64 × 64; slice thickness = 3.5 mm, voxel size = 3.1 × 3.1 × 3.5 mm^3^; and a total of 200 time points.

We classified and studied functional images with MRIcro software (www.MRIcro.com). Data were pre-processed with RESTplus V1.2 (http://www.restfmri.net) toolbox based on the MATLAB R2018b platform (28). Initially, the first ten functional volumes were excluded to acquire balanced measurement signals. Then, it was followed by form transformation (DICOM to NIFTI), slice timing, correction of head motion, Montreal Neurological Institute (MNI) space normalization (using T1 image unified segmentation), and re-sample data at 3 × 3 × 3 mm^3^ resolution, smoothing (full-width Gaussian kernel = 6 × 6 × 6 mm^3^), and also linear detrending and filtering (0.01–0.08 Hz) (29). In total, 11 of them were excluded due to head movement over 3-mm translocation or over 3° rotation toward each direction in scanning (5 in the LLD group and 6 in the NCs group). This study referenced Friston's 24 head-motion parameters as covariates in regressing the head motion effects. Linear regression was adopted for removing covariates for white matter, global mean signal, cerebrospinal fluid signal, and head motion. In addition, the PerAF approach indicates the ratio of frequency-domain in blood oxygen level-dependent signal in the resting-state to average signal strength for a specific period. Following the pre-processing, the PerAF method was computed (30).


(1) $$\begin{array}{r} P\text{er}AF=\frac{1}{\text{n}}\sum_{\text{i}=1}^{\text{n}}|\frac{{\text{x}}_{\text{i}}-\mu }{\mu }|\;\times \text{100} \end{array}$$

(2) $$\begin{array}{r} \mu =\frac{1}{\text{n}}\sum_{i=1}^{\text{n}}x_{i} \end{array}$$

where “X” represents the signal intensity of the time point, “*n*” refers to the total number of time points of the time course, and “μ” represents the mean value of the time course.

### Statistical Analysis

In this study, statistical analyses were conducted with SPSS 26.0 (SPSS, Chicago, IL, USA). The independent descriptive variables (age) were expressed as mean ± SD, whereas categorical variables (sex) were expressed as counts and percentages. Comparison for continuous variables was conducted by performing an independent *t*-test, and comparison for categorical variables was made with chi-square or Mann–Whitney *U* test. Statistical test differences between the LLD and NCs groups showed statistical significance with *P*-values < 0.05. The ‘Statistical Analysis' module of the RESTplus V1.2 toolbox was adopted to compare the average PerAF values of the LLD group and the NCs group. To maintain the balance between the two groups. Some covariates, such as age, gender, and education level, were regressed. For the preliminary comparison results of the average PerAF values between the two groups, the multiple-comparison correction based on the Gaussian random field theory (GRF, two-tailed, cluster-wise *p* < 0.05, voxel-wise *p* < 0.001) was further conducted (30). Previous studies reported that, compared with the Bonferroni correction and false discovery rate (FDR), GRF could reduce the false-positive rate and improve statistical power by utilizing the spatial information of fMRI data (31). Subsequently, the “Viewer” module displayed the brain regions with different perAF values between the two groups. Then, the brain regions with different PerAF values were further made into masks, and the PerAF values of altered brain regions of patients with LLD were extracted using the “Extract ROl Signals” module in RESTplus V1.2 toolbox for the next analysis. Here, we adopted the receiver operating characteristic (ROC) curves for identifying the presented PerAF method's ability to distinguish the two groups compared with the RBANS test. The correlations of the PerAF values for the region of interests (ROIs) with clinical symptoms (HAMD-17 and RBANS score) were assessed through Pearson correlation analysis.

## Results

### Demographics and Neuropsychologic Data

Upon removing patients with head motion above 3° rotation or 3 mm translocation (5 in the LLD group, 3 men and 2 women; 6 in the NCs group, 2 men and 4 women), 45 in the LLD group and 34 in the NCs group were eventually recruited for the current research. Patients with the LLD and NCs were aged 67.04 ± 4.51 and 65.12 ± 3.98, respectively. No differences in age, gender, and educational level could be observed. However, differences in the RBANS total score were observed in immediate memory, visuospatial/constructional, language, attention, and delayed memory between groups (*P* < 0.05). More details are illustrated in Table 1.

**Table 1:** Demographics and neuropsychologic data of the LLD group and the NCs group.

**Characteristic**	**LLD group**	**NCs group**	**Statistics**	***P-*** **value**
	**(*n* = 45)**	**(*n* =34)**	**t/x^**2**^**	
Sex (male/female)	15/30	16/18	1.530	0.216
Age (years)	67.04 ± 4.51	65.12 ± 3.98	1.976	0.052
Education (years)	9.47 ± 3.15	10.79 ± 3.22	1.837	0.071
RBANS total score	94.62 ± 12.62	119.65 ± 14.85	8.084	<0.001
Immediate memory	95.27 ± 12.12	113.18 ± 15.24	5.820	<0.001
Visuospatial/Constructional	97.09 ± 11.2	108.44 ± 9.38	4.769	<0.001
Language	94.58 ± 10.52	106.47 ± 5.95	5.913	<0.001
Attention	106.80 ± 13.51	123.26 ± 8.08	6.301	<0.001
Delayed memory	91.78 ± 11.1	105.32 ± 10.17	5.552	<0.001

*LLD, late-life depression; NCs, non-depressed controls; RBANS, repeatable battery for assessing neuropsychological status*.

### PerAF Differences

**Figure 1:**
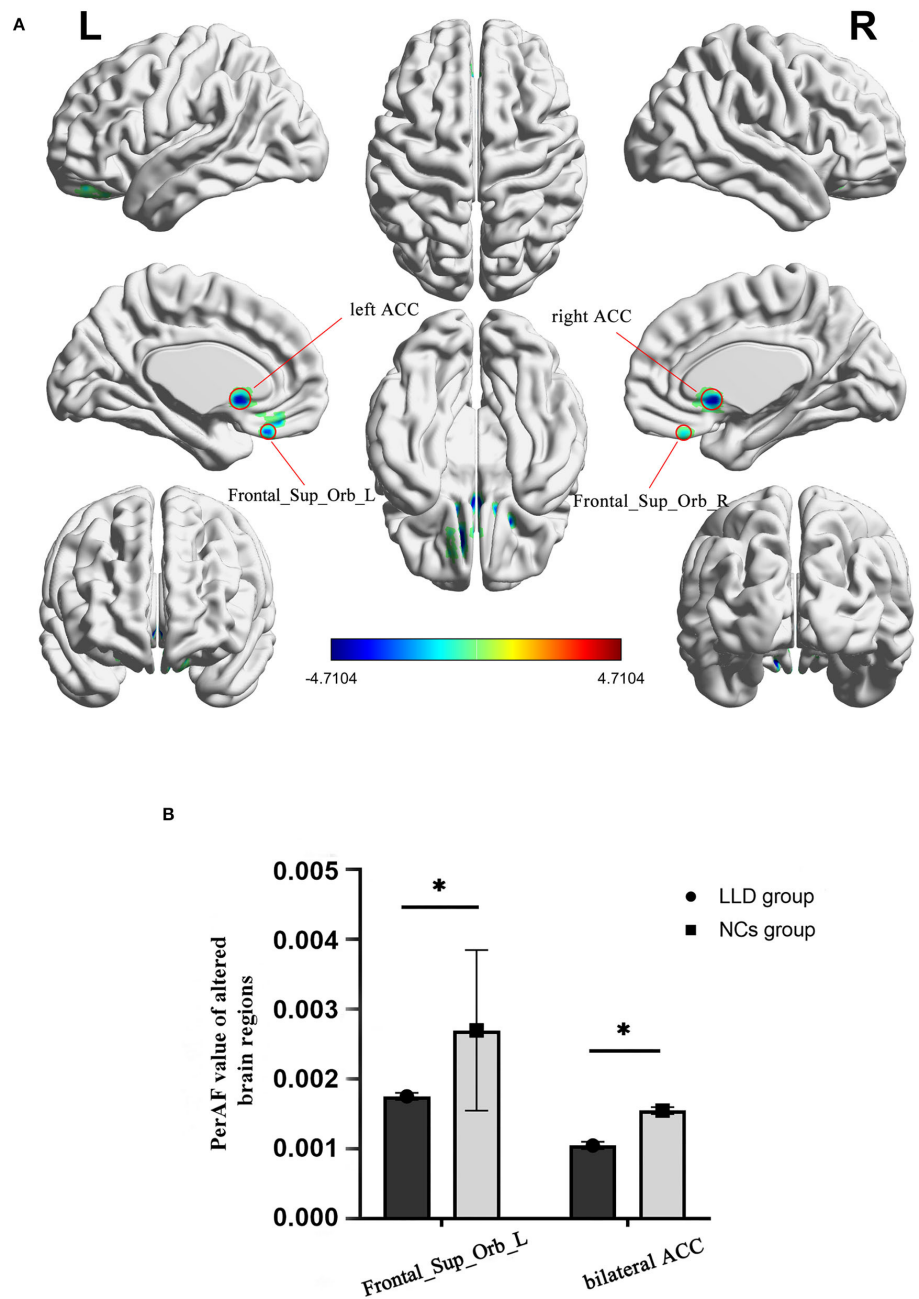
Altered brain regions in PerAF of the LLD group and the NCs group. **(A)** A comprehensive view; Blue color, decreased PerAF areas R, right; L, left; PerAF, percent amplitude of fluctuation; by contrast to the NCs group, the LLD group showed decreased PerAF differences in the bilateral superior frontal gyrus, orbital part (Frontal_Sup_Orb) [Brodmann area (BA) 11] and bilateral anterior cingulate cortex (ACC, BA24). **(B)** The mean PerAF signal value of bilateral Frontal_Sup_Orb and bilateral ACC between the two groups. Bilateral Frontal_Sup_Orb, bilateral superior frontal gyrus, orbital part; bilateral ACC, bilateral anterior cingulate cortex. *indicates that the difference between groups is statistically significant.

As shown in between-group statistical maps, by contrast to the NCs group, the LLD group showed decreased PerAF differences in the bilateral superior frontal gyrus, orbital part (Frontal_Sup_Orb) [Brodmann area (BA) 11], and bilateral anterior cingulate cortex (ACC, BA24) (Table 2, Figure 1A). Figure 1B displayed the mean PerAF signal value of altered brain regions between the two groups.

**Table 2:** Brain regions with significantly different PerAF values of the LLD group and the NCs group.

**Brain regions of peak coordinates**	**MNI Coordinates**			**BA**	**Number of Voxels**	**t-value**
	**X**	**Y**	**Z**			
LLD group < NCs group						
Bilateral Frontal_Sup_Orb	−9	39	−21	11	46	−4.2726
Bilateral ACC	6	15	−6	24	130	−4.7104

*Frontal_Sup_Orb_L, superior frontal gyrus, orbital part, this anatomical name originates from Automated anatomical labeling atlas (32); Bilateral ACC, bilateral anterior cingulate cortex; MNI, Montreal neurological institute; BA, Brodmann's area*.

### ROC Curve Analysis

**Figure 2:**
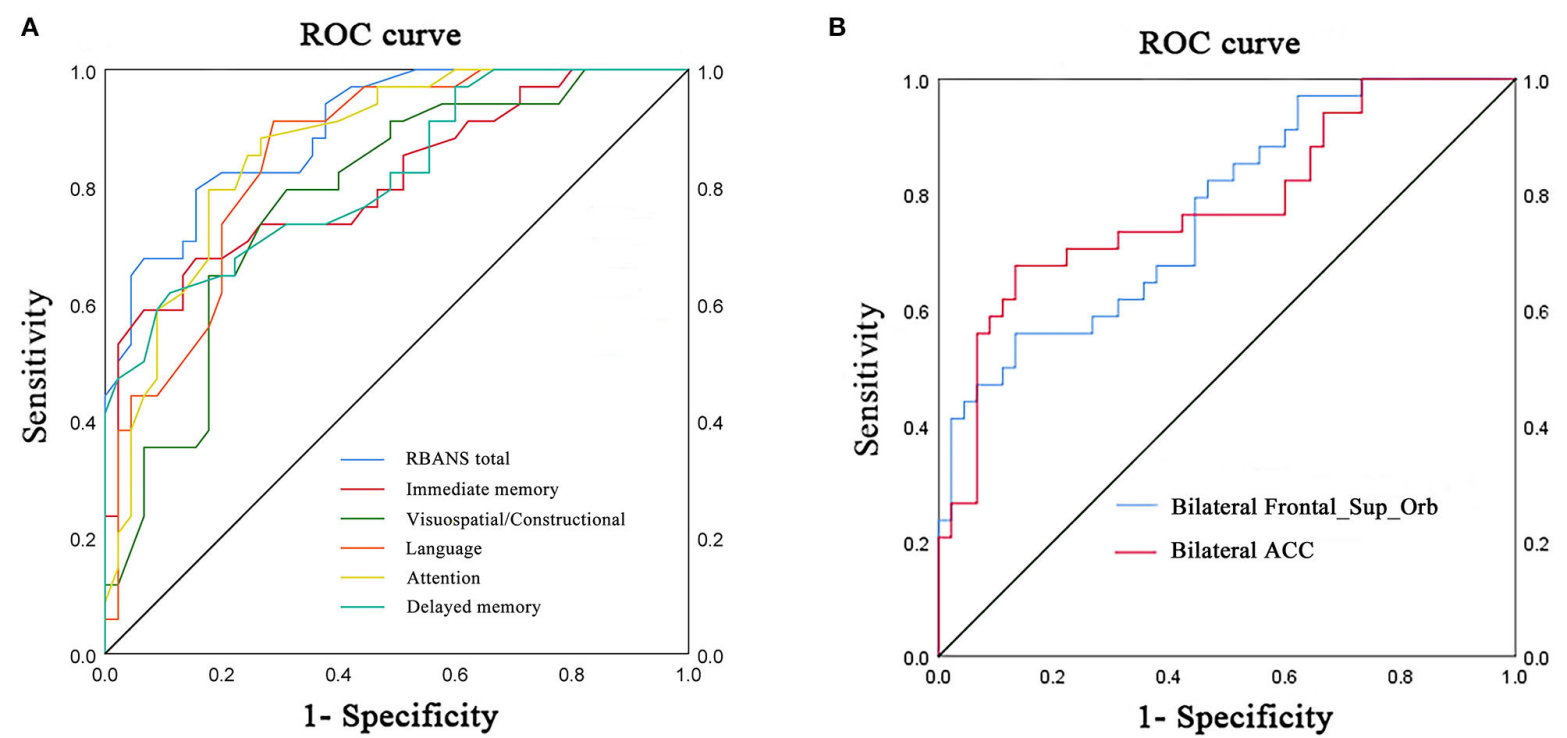
ROC curve analysis of RBANS test and PerAF values of altered brain regions in distinguishing LLD group from NCs group. **(A)** ROC curve analysis of RBANS total, immediate memory, visuospatial/constructional, language, attention, and delayed memory. **(B)** ROC curve analysis of bilateral Frontal_Sup_Orb and bilateral ACC. ROC, receiver operating characteristic; bilateral Frontal_Sup_Orb, bilateral superior frontal gyrus, orbital part; bilateral ACC, bilateral anterior cingulate cortex.

Receiver operating characteristic curve analysis and further diagnostic analysis were performed to evaluate the ability of the RBANS test and PerAF method in distinguishing the two groups, and higher area under curve (AUC) value indicated higher diagnostic accuracy. As for the RBANS test, AUC value for RBANS total score reached 0.899 (*P* < 0.001; 95% CI: 0.834–0.964, sensitivity: 0.794, specificity: 0.844), and AUC values of immediate memory, visuospatial/constructional, language, attention, and delayed memory were 0.809 (*P* < 0.001; 95% CI: 0.711–0.908, sensitivity: 0.676, specificity: 0.844), 0.781 (*P* < 0.001; 95% CI: 0.679–0.883, sensitivity: 0.794, specificity: 0.689), 0.852 (*P* < 0.001; 95% CI: 0.770–0.935, sensitivity: 0.912, specificity: 0.711) 0.866 (*P* < 0.001; 95% CI: 0.788–0.945, sensitivity: 0.882, specificity: 0.711), and 0.818 (*P* < 0.001; 95% CI: 0.725–0.911, sensitivity: 0.618, specificity: 0.889) (Figure 2A, Table 3), respectively. As a comparison, AUC value for bilateral Frontal_Sup_Orb reached 0.770 (*P* < 0.001; 95% CI: 0.667 −0.873, sensitivity: 0.559, specificity: 0.867), and AUC value of the bilateral ACC was 0.782 (*P* < 0.001; 95% CI: 0.676–0.887, sensitivity: 0.676, specificity: 0.867) (Figure 2B, Table 3).

**Table 3:** ROC curve analysis of RNANS test and PerAF values of altered brain regions in distinguishing the LLD group from the NCs group.

**Variables**	**AUC, 95%CI**	**Sensitivity, %**	**Specificity, %**	**Cut off Point^**a**^**
RBANS total	0.899 (0.834–0.964)	0.794	0.844	108
Immediate memory	0.809 (0.711–0.908)	0.676	0.844	122
Visuospatial/constructional	0.781 (0.679–0.883)	0.794	0.689	108
Language	0.852 (0.770–0.935)	0.912	0.711	102
Attention	0.866 (0.788–0.945)	0.882	0.711	121
Delayed memory	0.818 (0.725–0.911)	0.618	0.889	114
Bilateral Frontal_Sup_Orb	0.770 (0.667–0.873)	0.559	0.867	0.0047
Bilateral ACC	0.782 (0.676–0.887)	0.676	0.867	0.0016

a *Cut off point mean RBANS test score or PerAF signal value*.

### Correlation Analysis

**Figure 3:**
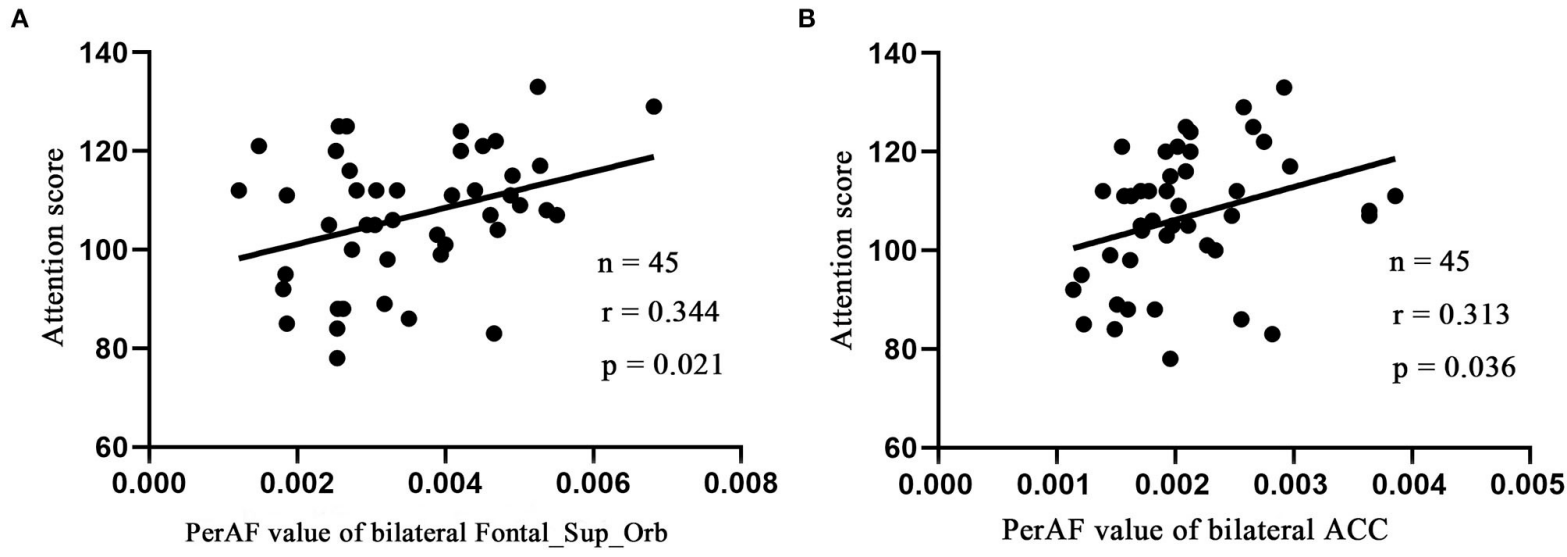
Pearson's correlation. Correlations were between attention score and bilateral Frontal_Sup_Orb **(A)**, bilateral ACC **(B)**. Bilateral Frontal_Sup_Orb, superior frontal gyrus, orbital part; bilateral ACC, bilateral anterior cingulate cortex.

Linear correlation analysis only observed the positive correlation between the attention score of RBANS test and the bilateral Frontal_Sup_Orb (*r* = 0.344, *p* = 0.021, *n* = 45, Figure 3A) and bilateral ACC (*r* = 0.313, *p* = 0.036, *n* = 45, Figure 3B) in the LLD group, respectively. It is worth noting that the current correlation results did not passed the multiple-comparison correction.

## Discussion

The current work performed a novel and more feasible method, namely, PerAF, which was also helpful in lowering the impact of BOLD signal strength, aiming to investigate the IBA in patients suffering from LLD. The findings indicated that the PerAF values of bilateral Frontal_Sup_Orb and bilateral ACC were significantly lower than those of the NCs. The RBANS test and PerAF values in the aforementioned altered brain regions exhibited a quite good discriminatory power with the AUC values in distinguishing the two groups.

Compared with the NCs group, patients with LLD exhibited poor-cognitive performance in all domains such as in the previous studies (33). Many hypotheses are present on the pathogenesis of LLD, one of which is depression executive dysfunction syndrome (DED). The main features of DED include decreased pleasure, intellectual disability, and lack of insight (34). In some cognitive tests, compared with the normal elderly, patients suffering from DED tend to score lower, such as language fluency, reaction inhibition, novelty problem solving, cognitive flexibility, and working memory, which are consistent with the destruction frontal-subcortical functional network. The disorder of subcortical structure, such as ACC, insula, and hippocampus, often occurs in patients with LLD, leading to abnormal IBA and projection to the pre-frontal cortex (PFC) (35).

Most researchers have demonstrated a declined metabolism within pre-frontal, parietal and temporal cortices and cingulate regions among patients suffering from major depression disorder (MDD) (36). According to the literature, frontal regions (ACC included) exert a key role in MDD-related ReHo pathophysiology. Similarly, our study also discovered that the IBA detected by the proposed PerAF method in bilateral Frontal_Sup_Orb and bilateral ACC of patients with LLD was decreased in contrast to the NCs group. Former research showed that, relative to controls, IBA of superior frontal gyrus among patients suffering from remitted geriatric depression was mitigated (37), and interestingly, the patients incorporated in our research suffered from moderate to severe depressive disorder. It suggests that the decreased IBA superior frontal gyrus does not recover with the relief of depression, which may be an inherent attribute of IBA in some patients suffering from LLD. However, these two studies are all cross-sectional designs.

Moreover, the current finding needs further support from large-scale longitudinal research. In addition, the ROC curve showed that the PerAF method was lower than the RBANS test in discriminatory power for differentiating the two groups. Indeed, the RBANS test, including immediate memory, visuospatial/constructional, language, attention, and delayed memory, is commonly used in the neuropsychological tests, it makes judgments based on the symptomatic characteristics of LLD, and the heterogeneity of the results varies greatly. The advantage of the PerAF method is that it is based on hemodynamics at the resting state. The results are objective and repeatable. From the observed perspective, it could be considered that bilateral Frontal_Sup_Orb and bilateral ACC both exhibited a quite good discriminatory power in differentiating the two groups, indicating that the PerAF method was potentially a neuroimaging indicator in identifying the individuals with depression among the elderly.

From the perspective of anatomical labeling atlas, Frontal_Sup_Orb is a part of the orbital frontal gyrus (OFC), which belongs to Brodmann area 11. Guo et al. (10) discovered that compared with the control group, the activation of the left superior temporal gyrus increased, while the activation of bilateral superior frontal gyrus decreased among first-episode, drug-naive patients with LLD (15). Similarly, an rs-fMRI study of late-life subthreshold depression (StD) revealed that, compared with the controls, subjects with StD displayed a lower ReHo value in the right OFC (38). OFC is considered to play a key role in the pathophysiology of depression (39). A study demonstrated that patients with LLD exhibited smaller OFC volumes and positively correlated with white-matter lesion volume (40). A study exhibited that compared with patients with LLD without odor identification (OI) impairment and normal controls, patients with LLD with OI impairment exhibited increased functional connectivity (FC) between the left OFC and left calcarine gyrus, between the left OFC and right lingual gyrus, between the right OFC and right rectus gyrus (41). Odor identification (OI) impairment increases the risk of Alzheimer's disease in patients with LLD (42). Hypoactivity in the superior frontal gyrus may partially result in non-response to initial antidepressants in patients with LLD (43). Reports on OFC indicate that abnormal resting-state activity in Frontal_Sup_Orb or OFC may be related to the persistence of depressive symptoms in some patients with LLD, which should also arouse the attention of the elderly with StD.

The ACC connects structurally and functionally with various brain areas, including the lateral pre-frontal cortex, OFC, parietal cortex, amygdala, superior temporal gyrus (STG), nucleus accumbens, hypothalamus, insula, raphe nucleus, and hippocampus (44, 45). In addition, the ACC is thought to play a crucial role in allocating attentional resources in situations of conflicting cognitive and emotional demands (46). One study revealed that patients with LLD in the depressed and remitted phases showed significantly smaller gray matter volume in the left ACC and left posterior STG than healthy subjects (47). In addition, they also discovered that remitted patients with LLD showed lower functional ACC-pSTG connectivity than healthy subjects and positively correlated with lower global function during remission. Liu et al. (48) applied a novel analytical method, named coherence-based regional homogeneity (Cohe–ReHo), to assess regional IBA during the resting state in 15 first-episode, treatment-naive patients with LLD and demonstrated that, compared with the healthy controls, the LLD group showed significantly decreased Cohe–ReHo in right ACC (48). Thus, our finding that decreased PerAF in the bilateral ACC is compatible with these previous studies. These findings suggest that functional alteration in ACC is deeply involved in depression.

Executive function processes, such as focusing attention, organizing, and strategizing, are representative symptoms of LLD, fully demonstrated to be maintained by frontotemporal brain regions (49). The superior frontal gyrus is conventionally considered the frontal eye field. However, functional research has revealed the major contribution of this region to executive function, working memory, and attention (50, 51). As a major function of cognition, attention is essential to perception, language, and memory. Individuals with MDD exhibited impairment in emotion and attention control. The regulation of emotion is vital for adaptive behavior in a social environment. The different strategies may be adopted to achieve successful emotion regulation, ranging from attentional control (e.g., distraction) to cognitive change (e.g., reappraisal). A study revealed that the OFC is involved in distinguishing presently relevant from previously relevant information and was activated for reappraisal only (52, 53).

In contrast, the attentional control condition is characterized by orienting attention away from the emotional stimulus to a cognitive task, the commitment of resources to the processing of this task, and the detection of potential conflicts between task processing and emotional activation. The dorsal portion of the ACC has been widely discussed as a major node in the attentional control network, for the monitoring of conflict between opposing activations (54). Loeffler et al. (55) thought that attention control and emotion regulation are conceptually similar and might share common mechanisms (55), which indicate that emotional disturbances among patients suffering from LLD can be ameliorated through interventions with target attention control.

In this study, the attention score of the RBANS test is positively correlated with the PerAF values in Frontal_Sup_Orb_L and bilateral ACC. During the cognitive assessment of the RBANS test, the Attention test contains two subtests, respectively, Digit Span Test and Symbol-Digit Coding Test. The former can reflect working memory capacity to a certain extent, while the latter is closely related to psychomotor speed and cognitive flexibility (56, 57), which also reflects the inherent complexity and hierarchy of advanced cognitive function. Attention, interference control, and working memory are essential components of executive function. Executive dysfunction is one of the representative symptoms of patients with LLD. Therefore, it can be speculated that in future intervention studies, if bilateral Frontal_Sup_Orb and bilateral ACC can be used as therapeutic targets, it may be conducive to improving the executive function of LLD.

However, the research has some limitations. First, the participants all come from or around Beijing, near our hospital, which decides the small sample size. Second, adequate attention must be paid to potential confounders, such as diabetes and hypertension, often co-morbid with LLD. Personality and special skills also affect resting-state hemodynamic fluctuations, thus, requiring further evaluation and control in future research. Third, changes in cognitive impairment severity result in confounding bias though average MMSE scores of the two groups are all >24. Finally, research has demonstrated that the neuroendocrine stress and subjective reactions resulting from MRI involvement affect brain functioning, which relates to adverse reactions, such as dizziness, phosphenes, and head ringing (58). To solve the existing problem, a several-minute habituation time is recommended for the participants by the scanner in MRI research before the actual examination, particularly, for female participants (59).

## Conclusion

Notwithstanding the limitations mentioned earlier, the research showed that changes of PerAF in bilateral Frontal_Sup_Orb and bilateral ACC are related to an increased risk of developing LLD. Moreover, the PerAF method could be used as an underlying sensitivity biomarker to identify the psychiatric disorders.

## Data Availability Statement

The raw data supporting the conclusions of this article will be made available by the authors, without undue reservation.

## Ethics Statement

The studies involving human participants were reviewed and approved by Beijing Anding Hospital Affiliated to Capital Medical University. The patients/participants provided their written informed consent to participate in this study.

## Author Contributions

CL and WP conceived and designed the research protocol. CL completed the data analyses. DZ and PM assisted with neuropsychological assessment and data processing. YR and XM checked the rs-fMRI data and revised the manuscript. All authors contributed to the article and approved the submitted version.

## Funding

The present work was supported by Beijing Municipal Science & Technology Commission (No. Z191100006619105).

## Conflict of Interest

The authors declare that the research was conducted in the absence of any commercial or financial relationships that could be construed as a potential conflict of interest.

## Publisher's Note

All claims expressed in this article are solely those of the authors and do not necessarily represent those of their affiliated organizations, or those of the publisher, the editors and the reviewers. Any product that may be evaluated in this article, or claim that may be made by its manufacturer, is not guaranteed or endorsed by the publisher.
